# Targeting the Gut–Bone Axis with Prebiotics, Probiotics, Synbiotics, and Postbiotics: From Mechanistic Plausibility to Clinical Bone Outcomes

**DOI:** 10.3390/nu18182967

**Published:** 2026-09-10

**Authors:** Sorina Ispas, Viviana Maggio, Adil Farooq Wali, Sirajunisa Talath, Syed Arman Rabbani, Bhoomendra A. Bhongade, Imran Rashid Rangraze, Shakta Mani Satyam, Ashot Avagimyan, Karolina Hoffmann, Ioannis Ilias, Anna Paczkowska, Mohamed El-Tanani, Manfredi Rizzo

**Affiliations:** 1 Department of Anatomy, Faculty of General Medicine, “Ovidius” University, 900470 Constanta, Romania; 2 School of Medicine, PROMISE Department of Health Promotion Sciences Maternal and Infantile Care, Internal Medicine and Medicinal Specialties, University of Palermo, 90133 Palermo, Italy; 3 RAK College of Pharmacy, RAK Medical and Health Sciences University, Ras Al Khaimah 11172, United Arab Emirates; 4 RAK College of Medical Sciences, RAK Medical and Health Sciences University, Ras Al Khaimah 11172, United Arab Emirates; 5 Department of Internal Diseases Propedeutics, Yerevan State Medical University, Yerevan 0025, Armenia; 6 Department and Clinic of Internal Diseases and Metabolic Disorders, Poznań University of Medical Sciences, 61-701 Poznan, Poland; 7 Department of Endocrinology, Diabetes and Metabolism, Elena Venizelou Hospital, GR-11521 Athens, Greece; 8 Department of Pharmacoeconomics and Social Pharmacy, Poznań University of Medical Sciences, 61-701 Poznan, Poland

**Keywords:** osteoporosis, gastrointestinal microbiome, small intestinal bacterial overgrowth, prebiotics, probiotics, synbiotics, postbiotics, gut–bone axis, bone remodeling, osteoimmunology

## Abstract

Osteoporosis is a major skeletal disorder. Increasing evidence suggests that gut microbiota-derived mechanisms may contribute to bone remodeling. The biological rationale for a gut–bone axis is well supported, but the clinical relevance of microbiome-targeted interventions remains uncertain. In this narrative review, we focus on prebiotics, probiotics, synbiotics, and postbiotics. We searched in the literature the progress from mechanistic observations to measurable skeletal outcomes in humans. We searched PubMed, Scopus, and Web of Science for literature published mainly between 2021 and 2026. We also included earlier studies when they were important for the mechanistic framework. The evidence was evaluated according to intervention type and outcome. We focused on bone turnover markers, bone mineral density, and the duration of exposure required for these outcomes to become clinically meaningful. Recent randomized trials and meta-analyses were also compared in a semi-quantitative synthesis to show where findings are consistent and where they remain conflicting.

## 1. Introduction

Osteoporosis is a global health burden characterized by reduced bone mass, deterioration of bone microarchitecture, skeletal fragility, and increased fracture risk. It is associated with significant morbidity, mortality, and healthcare costs worldwide. Although traditional risk factors, such as aging and menopause, are well established [1], additional systemic regulators, including the gut microbiota, may play an important role in bone homeostasis [2]. Gut microbiota may influence skeletal integrity through metabolic, immune, and endocrine pathways. The gut–bone axis concept describes the interaction between intestinal microorganisms, microbial metabolites, immune signaling, nutrient absorption and bone metabolism [3]. Microbial metabolites, including tryptophan-derived compounds, bile acids, and short-chain fatty acids (SCFAs) may influence osteoclast and osteoblast function. These pathways are involved in bone remodeling [4,5,6].

Dysbiosis, defined as an imbalance in gut microbial composition or function, has been associated with several chronic conditions, including osteoporosis. According to both experimental and clinical data, dysbiosis may compromise the intestinal barrier function and may impair calcium [7]. Chronic low-grade inflammation may promote bone resorption and contribute to the loss of bone mineral density [8]. The individuals who are more vulnerable to these alterations in the microbiome are postmenopausal women and the elderly [9], as well as those exposed to antibiotics [10] or high-fat diets [11].

Numerous reviews described molecular and cellular pathways related to the gut microbiome to bone remodeling. The rapid expansion of this literature has not been accompanied by a proportional increase in clinically actionable evidence. Important distinctions between preclinical mechanistic findings, observational human associations, and interventional evidence are frequently blurred, while methodological heterogeneity limits the reproducibility of reported microbial signatures. Another broad description of the gut–bone axis would provide limited additional value. This structured narrative review critically examines the evidence linking the gut–bone axis to osteoporosis. It distinguishes findings from experimental models from human observational and interventional studies and evaluates their current clinical relevance (Table 1).

This review focuses on microbiome-targeted interventions in osteoporosis, including prebiotics, probiotics, synbiotics, and postbiotics. It also addresses the bidirectional interaction between gut microbiota and osteoporosis pharmacotherapy [20,21] and the current validation status of microbiota-related biomarkers [22]. Emphasis is placed on strain- and substrate-specific effects, emerging postbiotic mechanisms, semi-quantitative comparison of clinical outcomes, and the largely unexplored microbiota–drug–bone relationship.

## 2. Materials and Methods

### 2.1. Review Design and Reporting Framework

This article was designed as a structured narrative review on the gut–bone axis in osteoporosis. The review was developed to bring together mechanistic, translational, and clinical evidence on gut microbiota, dysbiosis, small intestinal bacterial overgrowth, microbial metabolites, mineral metabolism, osteoimmunology, endocrine signaling, and microbiome-targeted therapeutic perspectives.

The reporting approach was guided by the Scale for the Assessment of Narrative Review Articles (SANRA), with particular attention to the clarity of the rationale, the aim of the review, the description of the literature search, appropriate citation of the literature, and balanced interpretation of the evidence [23]. This review was not conducted as a systematic review or scoping review. We did not perform a PRISMA flow diagram, formal risk-of-bias assessment, methodological quality assessment, or quantitative synthesis.

### 2.2. Search Strategy

We searched PubMed, Scopus, and Web of Science for articles published mainly between 2021 and 2026. The last search was performed on 10 August 2026. Only articles published in English were considered. Earlier articles were also included when they were important for the conceptual or mechanistic background of the gut–bone axis.

The search combined terms related to gut microbiota and bone health, including “gut microbiota”, “gut microbiome”, “gastrointestinal microbiome”, “dysbiosis”, “gut–bone axis”, “small intestinal bacterial overgrowth”, “SIBO”, “short-chain fatty acids”, “butyrate”, “bile acids”, “tryptophan metabolites”, “osteoporosis”, “osteopenia”, “bone mineral density”, “bone turnover markers”, “bone remodeling”, “osteoblasts”, “osteoclasts”, “osteoimmunology”, “incretin hormones”, “GIP”, “GLP-1”, “GLP-2”, “probiotics”, “prebiotics”, “synbiotics”, “postbiotics”, “drug-induced osteoporosis”, “pharmacomicrobiomics”, “secondary osteoporosis” and “fecal microbiota transplantation”. The database-specific search strategies and filters are presented in Supplementary Table S1.

### 2.3. Selection Criteria

We considered human studies, animal studies, experimental and translational studies, clinical and observational studies, systematic reviews, meta-analyses, and narrative reviews when they addressed gut microbiota, dysbiosis, SIBO, microbial metabolites, mineral absorption, immune regulation, endocrine pathways, bone mineral density, bone turnover markers, bone remodeling, fracture risk, or other osteoporosis-related outcomes.

Studies were included when they were relevant to at least one of the main themes of the review: gut microbiota composition, dysbiosis, SIBO-related mechanisms, short-chain fatty acids, bile acid signaling, tryptophan-derived metabolites, intestinal barrier dysfunction, osteoimmunology, calcium–vitamin D–magnesium homeostasis, incretin-related pathways, probiotics, prebiotics, synbiotics, postbiotics, fecal microbiota transplantation, or microbiome-related pharmacological approaches. Studies addressing the effect of osteoporosis medications on gut microbiota composition, or of drug-induced dysbiosis on skeletal outcomes, were also included.

We excluded conference abstracts without full text, letters, comments, editorials without original or mechanistic discussion, case reports without mechanistic relevance, non-English publications, and studies without a clear connection to gut microbiota, dysbiosis, SIBO, or skeletal outcomes. The inclusion and exclusion criteria are summarized in Supplementary Table S2.

### 2.4. Study Selection and Author Involvement

The literature search and initial organization of the material were coordinated by S.I., V.M., M.E.T., and A.F.W., with overall scientific supervision from M.R. Retrieved articles were screened for relevance by S.I., V.M., A.F.W., S.T., S.A.R., B.A.B., I.R.R., and S.M.S., according to the thematic focus of the review. Articles considered relevant were then reviewed thematically by authors with complementary expertise in anatomy, diabetes and metabolic diseases, biology, pharmaceutical chemistry, clinical pharmacy and pharmacology, internal medicine, pathology, endocrinology, metabolic disorders, pharmacoeconomics, and translational medicine. A.A., K.H., I.I., and A.P. contributed to the clinical, cardiovascular, endocrine, metabolic, and pharmacoeconomic interpretation of the evidence. Uncertainties regarding article relevance or thematic interpretation were resolved through discussion, with final oversight by M.E.T. and M.R.

### 2.5. Evidence Synthesis

The selected literature was synthesized narratively and organized into thematic sections. The synthesis focused on gut microbiota composition, dysbiosis, microbiota-derived metabolites, mineral metabolism, osteoimmunology, endocrine signaling, SIBO-related mechanisms, and microbiome-targeted preventive and therapeutic perspectives.

Because the included evidence came from different types of studies, we aimed to distinguish mechanistic plausibility from direct clinical evidence in humans. Preclinical findings were interpreted separately from human data whenever appropriate. Special attention was given to outcomes relevant to osteoporosis, including bone mineral density, bone turnover markers, bone remodeling, fracture risk, and potential therapeutic implications. Selected mechanisms and representative findings were summarized in tables to improve clarity and clinical interpretation.

For clinical interpretation, outcomes were considered according to a hierarchy of relevance. Mechanistic endpoints and microbiome changes were considered hypothesis-generating; mineral absorption and bone turnover markers were regarded as intermediate outcomes; sustained changes in bone mineral density were considered more clinically relevant structural outcomes; and fragility fracture reduction represented the most clinically meaningful endpoint. Findings based only on mechanistic or intermediate outcomes were not interpreted as evidence of established clinical benefit or fracture prevention.

Because this was a structured narrative review, no independent formal risk-of-bias or certainty-of-evidence assessment was performed. For randomized trials and meta-analyses included in the semi-quantitative harvest summary, we interpreted effect estimates in the context of the methodological limitations reported by the original authors, including heterogeneity, imprecision, publication bias, and certainty of evidence when available. Meta-analyses containing overlapping primary trials were not treated as independent bodies of evidence, and no additional pooling or quantitative weighting was performed.

The semi-quantitative harvest summary was restricted to randomized clinical trials and meta-analyses published between 2024 and 2026 in order to provide a focused overview of the most recent intervention evidence available at the time of the final search. Studies were selected for inclusion when they reported quantitative skeletal outcomes directly relevant to the clinical interpretation of microbiome-targeted interventions, including BMD or bone turnover markers. Because the included studies assessed different skeletal sites and used different outcome measures, effect estimates were not treated as directly interchangeable. Comparisons were therefore descriptive and were interpreted according to the specific anatomical site, endpoint, study population, and methodological limitations reported in the original publications.

## 3. Gut Microbiota and the Gut–Bone Axis: Essential Background

### 3.1. Microbiota Composition, Host Homeostasis, and Dysbiosis

The gastrointestinal tract hosts a diverse microbial population that contributes to the maintenance of the body’s physiological homeostasis [24]. The microbiota is composed of a complex community of microorganisms, including predominant bacteria, but also archaea, fungi and viruses. The four most represented phyla are *Proteobacteria, Actinobacteria, Bacteroidetes, and Firmicutes*. In most healthy adults, *Bacteroidetes* and *Firmicutes* represent more than 90% of the gut microbial population [25]. Beyond its microbial composition, the intestinal microbiota may modulate skeletal homeostasis [26].

Commensal microbes promote mucus production, stimulate antimicrobial peptide secretion, and strengthen tight junctions between epithelial cells [27].

Disruption of this balanced state, termed dysbiosis, predisposes individuals to gastrointestinal conditions and systemic pathologies, including cardiovascular disease, diabetes, obesity [28], and bone disorders, such as osteoporosis [9].

Gut microbiota dysbiosis can be defined as the pathological disruption in the composition, function, or metabolic activity of the intestinal microbial community [29]. Dysbiosis is marked by reduced diversity, depletion of beneficial taxa, as well as an overgrowth of pathogenic or opportunistic microorganisms [24]. In dysbiosis, butyrate production is diminished, which compromises the intestinal barrier function.

Western diets rich in refined sugar content and high fat diminish beneficial bacterial species [28].

Broad-spectrum antibiotics reduce microbial diversity, compromise resistance to colonization, while also facilitating the proliferation of opportunistic infections [10].

Aging is also associated with dysbiosis, fewer butyrate producers, as well as increased pro-inflammatory bacteria [30].

### 3.2. Short-Chain Fatty Acids, Mineral Absorption, and Intestinal Barrier Function

One of its key functions is the fermentation of dietary fibers and resistant starches into short-chain fatty acids—such as propionate, butyrate, and acetate [31].

Dysbiosis is associated with impaired bioavailability of minerals, including calcium and magnesium [32].

SCFAs and other microbial metabolites influence the relationship between pro-inflammatory Th17 cells and anti-inflammatory Tregs, a major determinant of bone resorption and formation [33].

In the case of intestinal barrier dysfunction, bacterial lipopolysaccharide can translocate into the circulation, driving chronic low-grade inflammation [34].

Antibiotic exposure exacerbates microbiota imbalance and impairs epithelial integrity [10].

### 3.3. From Dysbiosis to the Gut–Bone Axis

When this balance is disrupted, gut dysbiosis can augment cytokine production and osteoclast activation, contributing to osteoporosis and other inflammatory bone disorders [35].

This inflammatory state affects bone metabolic dynamics as it blocks osteoblast function and enhances osteoclast activity [35]. Modified microbial profiles and reduction in SCFA levels are associated with decreased bone mineral density [36]. Changes in gut microbiota composition were described in individuals with osteoporosis [15].

It also plays a key role in chronic disease pathophysiology, as well as the gut–bone axis.

The gut–bone axis represents an integrated network between the intestinal microbiome and its metabolites [3]. Multiple overlapping mechanisms—such as microbial metabolites, mineral metabolism, immune signaling, and endocrine pathways—mediate it.

## 4. Mechanistic Pathways Linking the Gut Microbiota to Bone

### 4.1. Role of Gut-Derived Metabolites

Direct or indirect roles in skeletal health are played by a variety of metabolites. For instance, butyrate, propionate, and acetate play an important role in the gut–bone axis [36].

Tryptophan derivatives (indoles, kynurenines) and polyphenol microbial metabolites exert systemic effects that may indirectly influence bone physiology. In experimental models, tryptophan-derived microbial metabolites signal through the aryl hydrocarbon receptor (AhR) and have been shown to influence osteoclast differentiation and intestinal barrier integrity [6]. Microbial transformation of dietary polyphenols, such as resveratrol, produces bioactive compounds with antioxidant and anti-inflammatory properties. This mechanism may indirectly contribute to osteoprotective effects [37].

### 4.2. Microbiota and Calcium and Vitamin D Metabolism

Vitamin D and calcium are cornerstones of skeletal integrity. The gut microbiota is related to vitamin D and calcium metabolism [38]. Microbial production of SCFAs modulates intestinal metabolic processes and contributes to host metabolic regulation [39]. The intestinal microbiome interacts with vitamin D metabolism and signaling. Vitamin D is essential for calcium absorption by influencing intestinal epithelial cells. Dysbiosis is associated with impaired vitamin D receptor (VDR) signaling [38]. New literature studies suggest that *Lactobacillus rhamnosus GG* administration may modulate the vitamin D/VDR signaling pathway [40]. Reduced levels of these bacteria have also been correlated with lower bone mineral density in the elderly population [15]. In a randomized clinical trial, supplementation with the specific strain *Lactobacillus reuteri* ATCC PTA 6475 was associated with reduced bone loss in older women with low bone mineral density [18].

Preclinical studies suggest that microbiota-modulating interventions may influence calcium retention and bone mass, although these findings cannot be directly extrapolated to human osteoporosis [41]. Experimental studies in germ-free mice demonstrate that the gut microbiota can regulate bone mass under controlled experimental conditions [12]. Fecal microbiota transplantation (FMT) is proposed as a potential therapeutic strategy for osteoporosis [42].

### 4.3. Osteoimmunology and Microbiota-Derived Metabolites in Bone Remodeling

Bone represents a dynamic tissue that is constantly remodeled by osteoclast-mediated resorption and osteoblast-mediated formation. This process is regulated by the immune system, which is known as osteoimmunology [35]. The gut microbiota is an essential component of the above-mentioned immune-bone mechanism [3].

Dysbiosis promotes systemic inflammation by increasing intestinal permeability and the translocation of bacterial antigens—such as LPS [43]. These antigens stimulate immune cells to release pro-inflammatory cytokines (TNF-α IL-6, and IL-1β), while also stimulating osteoclast-mediated bone resorption [35]. On the other hand, commensal microbes and their metabolites foster anti-inflammatory pathways, particularly through Treg expansion and IL-10 production, favoring bone formation [13].

Experimental evidence indicates that SCFAs can modulate osteoimmunological pathways, particularly through effects on regulatory T cells and the Th17/Treg balance. They stimulate the proliferation of Treg cells [13], increase their number, and stop the inflammation caused by Th17 cells [44]. In the ageing population, osteoporosis and inflammatory bone disorders, are associated with immune dysregulation and chronic low-grade inflammation [8]. This state is characterized by an imbalance between Th17 cells and Tregs, related with bone loss [44]. In ovariectomized rodent models, microbiota-related interventions have been shown to influence immune balance, reduce osteoclastogenesis, and attenuate bone loss. These findings provide mechanistic support but should not be interpreted as evidence of clinical efficacy in human osteoporosis [45].

Bacterial metabolites also affect the functions of osteoblasts. For instance, butyrate may stimulate Wnt/β-catenin signaling in osteoblasts, contributing to bone formation [13]. Bile acids and tryptophan metabolites additionally modulate osteoclast precursor development through AhR signaling [6].

### 4.4. The Butyrate-PTH Axis: Microbiota-Dependent Endocrine Control of Bone Anabolism

The butyrate–PTH pathway described in this section is supported predominantly by experimental evidence from murine models. Although these studies provide a detailed mechanistic framework linking microbial metabolites, immune regulation, and PTH-induced bone formation, direct evidence demonstrating the same pathway in humans with osteoporosis remains limited.

In experimental models, butyrate has been identified as a mechanistic link between gut microbial metabolism and osteoanabolic signaling [13]. Short- chain fatty acid signaling through GPR41/43 receptors are also described to be implicated in the regulation of bone mass [46]. Increased levels of butyrate, whether through probiotic administration, for example, *Lactobacillus rhamnosus GG* or direct supplementation, expand regulatory T (Treg) cells in both the bone marrow and the intestine. Regulatory T cells interact with BM CD8+ T cells to upregulate the expression of Wnt10b, a potent bone anabolic ligand [13]. Tregs facilitate the assembly of an NFAT1-SMAD3 transcription complex within the CD8+ T cells. This complex binds directly to the Wnt10b promoter to drive its transcription. Subsequently, Wnt10b activates Wnt/β-catenin signaling in osteoblast lineage and stromal cells, stimulating their differentiation and survival to promote bone formation [13].

In experimental models, the gut microbiota and butyrate act as a critical permissive cofactor for the bone anabolic response of Parathyroid Hormone (PTH). In the absence of an intact gut microbiota and insufficient butyrate production, PTH is unable to stimulate trabecular bone formation [14].

PTH requires butyrate to expand the population of Tregs in the bone marrow. While PTH itself does not directly target the cells that induce Treg differentiation, butyrate provides the necessary stimulus to allow this expansion to occur. The effect of butyrate is mediated through the activation of the SCFA receptor GPR43 on dendritic cells (DCs). Butyrate-stimulated DCs then support the differentiation of naïve CD4+ T cells into regulatory T cells. In mice lacking GPR43, PTH fails to expand Tregs and cannot stimulate bone formation [14].

Once expanded by the combined action of butyrate and PTH, Tregs stimulate CD8+ T cells in the bone marrow to produce the osteogenic Wnt ligand, Wnt10b. Tregs achieve this by silencing CD28 signaling in CD8+ T cells, which shifts the association of the transcription factor NFAT to SMADs (instead of AP-1), thereby activating the Wnt10b promoter [14].

The bone anabolic activity of PTH requires two independent and parallel pathways to function simultaneously:

1. PTH acts directly on osteocyte receptors. This interaction blocks Wnt inhibitors like sclerostin [14].
2. The microbiota/butyrate/Wnt10b pathway, which provides positive osteogenic stimulus. In murine models, PTH cannot stimulate new bone formation if the microbiota-dependent pathway is absent, even if the direct osteocyte pathway is active [14].

The signaling between the gut and bone is regulated by two primary microbial pathways: the Tryptophan-AhR axis, which focuses on intestinal barrier integrity and anti-inflammatory signaling [6], and the butyrate-PTH axis, which focuses on immune-mediated bone formation [14].

Microbial metabolism of dietary tryptophan (Trp) by gut bacteria produces indole derivatives. These metabolites act as ligands for the Aryl Hydrocarbon Receptor (AhR) in the intestinal epithelium [47]. Activation of AhR modulates β-catenin-dependent signaling pathways, which may increase the expression of tight junction proteins (E-cadherin, ZO-1) [48]. A restored intestinal barrier reduces the translocation of LPS into the circulation, reducing systemic inflammation [6].

The restored intestinal barrier favors the polarization of intestinal macrophages from a pro-inflammatory M1 phenotype to an anti-inflammatory M2 phenotype. These anti-inflammatory M2 macrophages secrete high levels of Interleukin-10 (IL-10), which reaches the circulation and the bone marrow [49]. In the bone marrow, IL-10 inhibits osteoclastogenesis (reducing bone resorption) [50].

The short-chain fatty acid (SCFA) butyrate, produced by microbial fermentation of dietary fibers acts as a critical cofactor for the bone anabolic effects of Parathyroid Hormone (PTH). Butyrate signals via the GPR43 receptor on dendritic cells (DCs) to facilitate the expansion of Regulatory T cells (Tregs) in the bone marrow [14].These Tregs interact with CD8+ T cells, inducing them to produce the osteogenic ligand Wnt10b [14]. Wnt10b activates Wnt signaling in osteoblasts, promoting their proliferation and survival, which is essential for PTH to stimulate new bone formation [14].

SCFAs like acetate also regulate bone architecture by influencing the differentiation of mesenchymal stem cells (MSCs) via Gpr41/43 receptors [46]. While excessive acetate can favor adipogenic differentiation over osteogenic differentiation [49], a balanced microbial environment ensures these metabolites support skeletal homeostasis [36].

Taken together, the butyrate–PTH–Treg–Wnt10b pathway is well characterized experimentally, particularly in murine models, but its relevance to human osteoporosis and to the clinical response to PTH-based therapy has not yet been established.

### 4.5. Bile Acid Signaling in Bone Remodeling

Current evidence for FXR- and TGR5-mediated skeletal effects is derived predominantly from cellular and animal models, whereas direct clinical evidence linking these pathways to osteoporosis outcomes in humans remains limited.

The role of bile acids and their receptors in bone metabolism involves specific cellular targets and well-defined intracellular signaling pathways that maintain skeletal health. Bile acids interact with several key cell types involved in bone remodeling. Activation of the farnesoid X receptor (FXR) in pluripotent MSCs (such as the C3H10T1/2 line) promotes their differentiation into the osteoblastic lineage while reciprocally inhibiting adipogenesis [5].

FXR is endogenously expressed in calvaria and bone marrow cells, with expression levels increasing progressively during osteoblastic differentiation [5]. FXR signaling is implicated in the regulation of bone metabolism and protection against bone loss [51]. The membrane bile acid receptor TGR5 is also expressed in osteoblasts, where its activation induces differentiation and mineralization [52].

Both FXR and TGR5 are expressed in osteoclast precursors (BMMs, or bone marrow-derived macrophages). Signaling through these receptors directly suppresses osteoclastogenesis [51].

Activation of FXR or TGR5 blocks DNA-binding activity and NF-κB nuclear translocation and while reducing the expression of c-Fos and NFATc1, factors critical for osteoclast formation [51].

In murine models, FXR deficiency increases the RANKL/OPG ratio, whereas its activation helps maintain this balance to limit bone resorption [5].

### 4.6. Endocrine Pathways Linking Gut and Bone

Microbiota-derived metabolites interact with hormonal systems that control the gut–bone axis and bone metabolism. The growth hormone (GH)/insulin-like growth factor 1 (IGF-1) axis is modulated by the bacterial composition of the gut microbiota. IGF-1 is essential for osteoblast proliferation and bone matrix synthesis. Thus, the gut microbiota has an indirect role in promoting bone formation [53].

The parathyroid hormone (PTH) pathway regulates calcium and phosphate metabolism, and it is also influenced by the gut microbiota. SCFAs produced by intestinal bacteria modulate the anabolic effects on bones. Experimental models demonstrate that SCFAs augment the osteoanabolic response to intermittent parathyroid hormone (PTH) treatment, indicating that microbial metabolites function as cofactors in endocrine bone modulation [14].

The sex steroid axis interacts with the microbiota, which forms an additional regulatory layer in bone turnover and skeletal health [54]. In menopause, estrogen deficiency alters gut microbial diversity; it reduces SCFA levels and also increases permeability, leading to enhanced osteoclast activity [55]. Adding estrogen supplementation can partially restore microbial balance, which suggests bidirectional regulation. The “microgenderome” concept has been suggested to describe how sex hormones and microbiota interact in order to influence disease risk, including osteoporosis [55].

Gut hormones, for example, incretin hormones glucagon-like peptide (GLP-1 and GLP-2) and GIP (Glucose-dependent insulinotropic polypeptide), peptide YY and ghrelin, are influenced by microbial metabolites and dietary inputs. These hormones regulate energy balance but also exert anabolic effects on bone by inhibiting osteoclastogenesis and stimulating osteoblast differentiation [56]. Glucagon-like peptide (GLP-1, GLP-2) and glucose-dependent insulinotropic polypeptide (GIP) influence bone resorption through their receptors. GIP exerts effects on bone formation [56].

GIP regulates bone remodeling through a dual function, exhibiting both anti-catabolic and potentially anabolic properties [57]. The anti-resorptive effects involve multiple signaling pathways that collectively limit the nuclear factor of activated T cells-1 (NFATc1) and nuclear translocation of nuclear factor-kB (NF- kB) activation within osteoclast precursors [57]. Endogenous GIP is responsible for postprandial suppression of bone resorption. A reduction in bone turnover markers was observed [58].

The GIP analogue (D-Ala2) GIP may inhibit inflammatory bone resorption by suppressing the production of proinflammatory cytokines TNF-α (in macrophages) and RANKL (in osteoblasts). There is also evidence of a direct effect on osteoclast formation [59].

Experimental models suggest that GIP signaling contributes to the maintenance of skeletal architecture [60]. Mice lacking GIP develop osteopenia [61]. Restoration or pharmacological activation of GIP signaling improves trabecular structure, which supports the hormone’s osteogenic role [59]. In short-term clinical investigations, GIP was found to transiently increase procollagen type 1 *N*-propeptide (P1NP). In vitro studies have shown that GIP increases alkaline phosphatase (ALP) activity and P1NP, which promotes osteoblast proliferation and differentiation [57].

Enteroendocrine K cells produce GIP in the duodenum and jejunum [62]. The relationship between the intestine, GIP, and bone is part of the gut–bone axis concept [63].

The main gut-derived hormones and microbiota-related signals involved in bone remodeling are summarized in Table 2.

GIPR activation in osteoblasts stimulates PI3K-Akt and cAMP-PKA signaling pathways, which are involved in reducing caspase-3/7 activation and decreasing apoptosis [57].

These mechanisms show that GIP promotes a favorable balance for bone mass maintenance by preserving osteoblast survival [57] and decreasing bone resorption [58]. The potential skeletal effects of GIPR agonism remain an area of investigation, and current evidence is insufficient to establish GIPR agonists as therapies for osteoporosis or fracture prevention [60].

L-cells secrete glucagon-like peptide-1 (GLP-1), glucagon-like peptide-2 (GLP-2), and peptide YY (PYY), a process strongly influenced by nutrient intake [56].

Both GLP-1 and GLP-2 have been associated with reduced bone loss [56]. GLP-1 has a dual function, exhibiting anabolic and anti-resorptive effects [64]. The GLP-1 receptor (GLP-1R) is detected in mouse osteoblast-like cells (MC3T3-E1) [65], bone marrow mesenchymal stem cells (BMSCs) [56], and immature human osteoblastic cell lines, including MG-63 and TE-85 [60].

Evidence across these endocrine pathways spans in vitro experiments, animal models, short-term human physiological studies, and a limited number of clinical investigations. Therefore, mechanistic effects observed at the cellular or animal level should not be assumed to represent established therapeutic effects in human osteoporosis.

Further long-term studies, particularly performed on humans, could contribute to a better understanding of the molecular mechanisms and enhance the safety of GIPR, GLP-1R, and GLP-2R agonists as possible treatments for osteoporosis.

## 5. The Impact of Gut Microbiota Dysbiosis on Bone Health

### 5.1. Preclinical Evidence

Germ-free mice are devoid of intestinal microbiota. In the study by Sjögren et al., germ-free mice exhibited increased bone mass and reduced osteoclast activity compared with conventionally raised mice. Colonization with a normal gut microbiota normalized bone mass, supporting a regulatory role of the gut microbiota in skeletal homeostasis [12].

Probiotic supplementation with *Lactobacillus rhamnosus GG* may modulate the gut microbiota composition and host signaling pathways [40]. SCFAs modulate the Th17/Treg balance, and suppress osteoclastogenesis [13].

After receiving fecal transplantation of microbiota from healthy donors, bone mass is restored [66].

### 5.2. Human Evidence

Xie et al. (2025) reported that reduced microbial diversity, depletion of Bifidobacterium and Lactobacillus, and expansion of pro-inflammatory species, for example, Enterobacteriaceae, have been associated with low bone mineral density (BMD) in both, women and men [67].

Li et al. (2024) reported alterations in gut microbial composition and fecal short-chain fatty acid profiles in women with postmenopausal osteoporosis. Reduced abundance of selected bacterial taxa and lower propionic acid concentrations were associated with lower bone mineral density [16].

Therapeutic interventions, such as probiotic supplementation, have been reported to reduce bone turnover markers [19] and improve bone mineral density in elderly populations [18].

Representative quantitative findings from key human and preclinical studies are summarized in Table 3 to provide a more clinically interpretable overview of the magnitude of microbiota-related effects on bone outcomes.

**Table 3:** Representative quantitative findings from key studies on the gut–bone axis.

Study	Population/Model	Exposure/Intervention	Main Outcome	Reported Quantitative Finding	Interpretation	Reference
Nilsson et al. (2018)	Older women with low BMD	*Lactobacillus reuteri* ATCC PTA 6475 for 12 months	Tibial volumetric BMD/bone loss	Tibial total vBMD decreased by −0.83% with L. reuteri versus −1.85% with placebo at 12 months (ITT); between-group mean difference 1.02 percentage points (95% CI 0.02–2.03).	Supports strain-specific antiresorptive potential	[18]
Vanitchanont et al. (2024)	Postmenopausal women with osteopenia	Multispecies probiotic supplementation for 12 weeks	Serum CTX	Significant reduction in serum CTX compared with baseline; no significant change in the placebo group	Supports a short-term antiresorptive effect	[19]
Rettedal et al. (2021)	Postmenopausal women with osteoporosis/osteopenia	Observational microbiome profiling	Taxonomic/functional microbiome differences	Distinct taxonomic and functional pathway differences were identified between women with osteoporosis/osteopenia and controls	Supports an association between microbiome alterations and low BMD, but not causality	[15]
Liu et al. (2026)	Patients with osteoporosis	*Limosilactobacillus fermentum* CCFM1126	BMD, serum calcium, 25(OH)D, gut microbiota	Significant increases in BMD, serum calcium, and 25(OH)D were reported after probiotic supplementation	Supports a potential beneficial skeletal effect of CCFM1126, requiring confirmation in independent clinical populations.	[68]
Resciniti et al. (2026)	114 early postmenopausal women	*Lactiplantibacillus plantarum* DSM15312/DSM15313 + *Lacticaseibacillus paracasei* DSM13434 for 12 months	Distal tibia volumetric BMD	Greater decline in distal tibia total vBMD versus placebo: −3.7 mg HA/cm^3^; 95% CI −6.9 to −0.4; *p* = 0.027	Does not support a generalized skeletal benefit of probiotic supplementation	[69]

In postmenopausal women, estrogen deficiency is associated with gut microbiota imbalance, increases intestinal permeability and promotes systemic inflammation, all of which drive osteoclast activity [70]. Elderly populations with reduced bacterial diversity are also at a higher risk of fractures, suggesting that age-related dysbiosis is an important determinant of osteoporosis progression [17].

Most clinical studies on the gut–bone axis have focused on postmenopausal women, whereas male osteoporosis remains comparatively understudied [71].

Experimental evidence suggests that bone loss associated with sex steroid deficiency is microbiota dependent, supporting the biological plausibility of sex-specific gut–bone interactions [72].

Future studies should include sex-stratified analyses and dedicated male cohorts rather than extrapolating predominantly female-derived data to male osteoporosis [71].

### 5.3. Small Intestinal Bacterial Overgrowth and Bone Deterioration (Osteoporosis)

SIBO may be associated with nutritional and body-composition abnormalities that could have implications for skeletal health [73]. Direct clinical evidence linking SIBO to osteoporosis remains limited.

An important challenge in interpreting the SIBO-bone link is the lack of standardized diagnostic criteria [74,75]. Existing studies have relied on different breath-test substrates, most commonly glucose and lactulose. They have also used different hydrogen- and methane-based positivity thresholds [76,77]. In addition, variation in sampling protocols, including substrate dose and test duration, can influence the number of positive results and their interpretation. Consequently, patient classification may differ substantially across studies. This heterogeneity undermines comparability, limits reproducibility, and weakens clinical applicability [74,75]. In the context of osteoporosis, such diagnostic uncertainty may affect both prevalence estimates and the interpretation of nutrient malabsorption-related skeletal risk [75,77]. Future studies should adopt harmonized diagnostic standards and report, at minimum, the substrate used, the gas profile assessed, positivity thresholds [76,77], test duration [75], and the relevant gastrointestinal clinical context [77].

**Figure 1:**
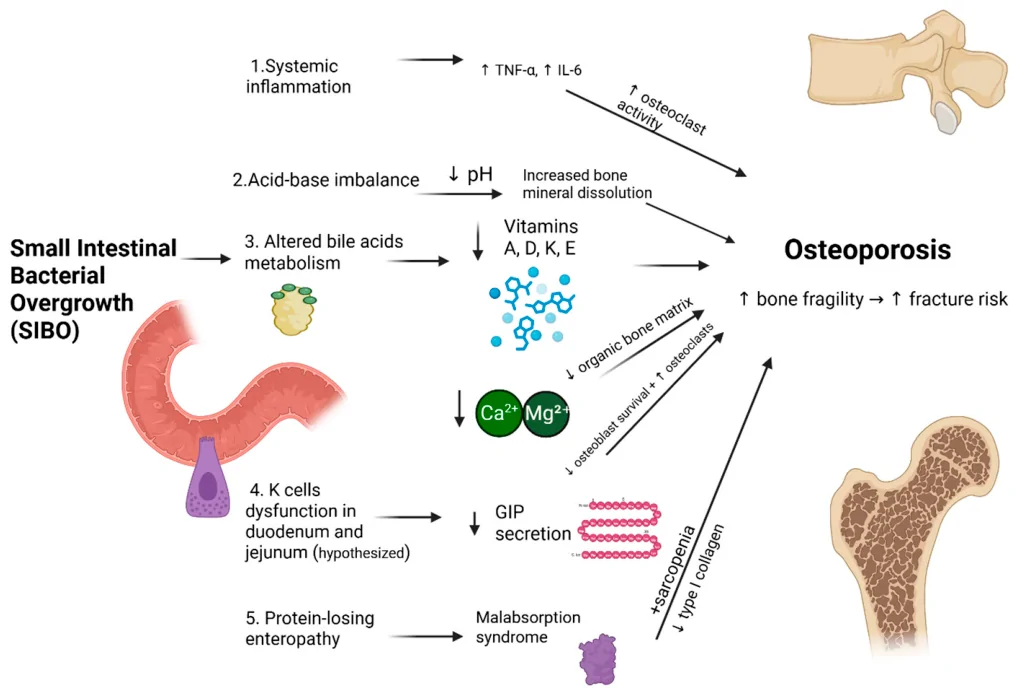
Mechanistic pathways linking small intestinal bacterial overgrowth (SIBO) to impaired bone metabolism and osteoporosis. Arrows indicate the proposed direction of influence among SIBO-related mechanisms, intermediate inflammatory, metabolic, and malabsorptive disturbances, and impaired bone metabolism leading to osteoporosis; they do not imply an established causal relationship. Created in BioRender. Ispas, S. (2026) https://BioRender.com/fxg8d94, Accessed on 13 August 2026. Figure 1 illustrates possible pathophysiological pathways through which SIBO may contribute to bone deterioration. These mechanisms include systemic inflammation, acid-base imbalance, bile acid disruption, malabsorption syndromes, K-cell dysfunction with reduced GIP secretion, and multiple nutrient deficiencies. Collectively, these proposed mechanisms may adversely influence bone remodeling and could contribute to skeletal fragility in susceptible patients; however, a causal relationship between SIBO and osteoporosis has not been established. **Abbreviations:** Ca^2+^, calcium; Mg^2+^, magnesium; SIBO, small intestinal bacterial overgrowth.

The proposed mechanisms linking SIBO to impaired bone metabolism and osteoporosis are summarized in Figure 1.

Body composition parameters, such as mineral bone content, may show an inverse correlation with hydrogen production in patients with SIBO. It is recommended to perform a body composition analysis (such as Bioelectrical Impedance Analysis—BIA) in patients with SIBO. This analysis serves as a basic tool to assess and monitor anthropometric parameters, since SIBO treatment often involves restrictive diets that could potentially worsen undernutrition [73]. In patients with unexplained osteoporosis, SIBO and other gastrointestinal diseases should be considered and carefully evaluated.

**Figure 2:**
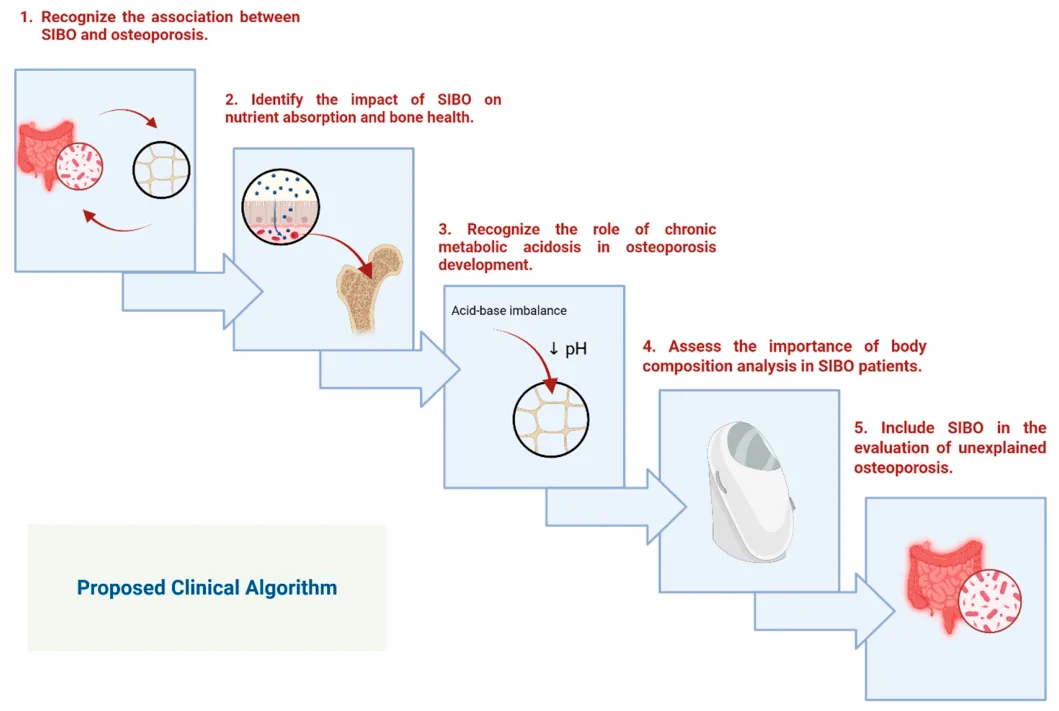
Key clinical considerations linking SIBO to impaired bone health and osteoporosis. Created in BioRender. Ispas, S. (2026) https://BioRender.com/fxg8d94, Accessed on 13 August 2026. Figure 2 highlights the proposed algorithm as a strategy for monitoring patients with unexplained osteoporosis but suspected SIBO. **Abbreviations:** SIBO, small intestinal bacterial overgrowth. Figure created with BioRender.com.

A practical clinical approach for evaluating patients with unexplained osteoporosis and suspected gastrointestinal dysfunction is presented in Figure 2.

Gut assessment may be considered in patients with unexplained osteoporosis, persistent gastrointestinal symptoms, or clinical features suggestive of malabsorption [77].

In such cases, the initial evaluation may include targeted gastrointestinal history, nutritional assessment, and consideration of conditions such as SIBO or other disorders affecting nutrient absorption [73].

Microbiota-targeted interventions should not be applied routinely to all patients with osteoporosis, but may be considered selectively when gastrointestinal dysfunction is suspected to contribute to skeletal fragility [42].

We propose this algorithm as a structured, citable clinical evaluation pathway for patients with unexplained osteoporosis and suspected gastrointestinal dysfunction, intended as a basis for future prospective validation [78].

Most microbiota–bone studies focus on the colon, where microbial fermentation of nondigestible carbohydrates generates short-chain fatty acids that exert systemic immunometabolic and skeletal effects [79].

The small intestine is more directly involved in micronutrient absorption, including pathways relevant to calcium and magnesium homeostasis [32].

Small-intestinal function is also closely linked to enteroendocrine signaling relevant to bone metabolism, including incretin-related pathways [56].

## 6. Prebiotics in Gut–Bone Axis

Diet modulates the gut microbiota and reshapes microbial composition [80]. High-fiber diets are associated with increased beneficial bacterial taxa, for example, Lactobacillus and Bifidobacterium, which produce short-chain fatty acids [81]. Inulin, a prebiotic non-digestible food ingredient, and fructooligosaccharides stimulate the growth of beneficial bacteria [82]. In experimental models, this microbial modulation may improve calcium absorption and bone mineral density (BMD) [82].

The habitual intake of fermented dairy products is associated with normal gut microbial diversity and may decrease fracture risk [83]. Polyphenols found in fruits, tea, and wine undergo microbial metabolism into bioactive derivatives with antioxidant and anti-inflammatory effects, indirectly supporting bone integrity [37].

### 6.1. Clinical Trial Evidence: Dose, Duration, and Population

Direct clinical evidence for inulin-type fructans on skeletal outcomes comes primarily from calcium-absorption and bone-accretion studies rather than fracture or BMD trials in osteoporotic populations. In pubertal adolescents randomized to 8 g/day of a mixed short- and long-chain inulin-type fructan (Synergy 1) versus maltodextrin placebo, calcium absorption was significantly greater in the fructan group at 8 weeks (difference: 8.5 ± 1.6%; *p* < 0.001) and at 1 year (difference: 5.9 ± 2.8%; *p* = 0.04), with a corresponding increase in whole-body bone mineral content (difference: 35 ± 16 g; *p* = 0.03) and whole-body bone mineral density (difference: 0.015 ± 0.004 g/cm^2^) after 1 year [84].

In postmenopausal women, a 6-week double-blind, placebo-controlled crossover trial of oligofructose-enriched inulin reported increased intestinal calcium and magnesium absorption alongside a short-term decrease in a marker of bone resorption, though no clear pattern emerged across all bone turnover markers [85].

In a randomized, double-blind, placebo-controlled trial involving postmenopausal women with a history of Roux-en-Y gastric bypass, Wu et al. administered 20 g/day of soluble corn fiber for 2 months and assessed fractional calcium absorption using a dual stable-isotope method. The study also evaluated calciotropic hormones, bone turnover markers, and fecal microbial composition, providing a more integrated assessment of the prebiotic–microbiome–bone relationship [86].

These studies suggest that prebiotic effects on bone-related outcomes may depend on the substrate used, baseline nutritional and gastrointestinal status, treatment duration, and the population studied. Available trials have evaluated intermediate outcomes such as mineral absorption or bone turnover rather than fracture risk or long-term BMD changes in patients with established osteoporosis.

### 6.2. Critical Appraisal

The available clinical evidence for prebiotics and bone health remains heterogeneous. Existing studies differ substantially in the prebiotic substrate used, dose, intervention duration, population, and skeletal outcome assessed. Early trials with inulin-type fructans reported improvements in calcium absorption and selected measures of bone mineralization [84,85]. More recent evidence using soluble corn fiber in postmenopausal women did not demonstrate consistent improvements in calcium absorption or bone turnover markers [86].

Another important limitation is the choice of study endpoints. Most trials have focused on calcium or magnesium absorption and short-term bone turnover markers, while relatively few have evaluated sustained changes in bone mineral density. Fracture outcomes have not been adequately studied. Moreover, the available evidence comes from markedly different populations, including adolescents and postmenopausal women with altered gastrointestinal anatomy, limiting direct extrapolation to patients with established osteoporosis. For clinical interpretation, these populations should be considered separately rather than as a single continuum of osteoporosis treatment evidence. Studies in adolescents primarily inform skeletal growth and mineral accrual, whereas studies in postmenopausal women with osteopenia or low BMD address early bone loss, and studies in patients with established osteoporosis are more directly relevant to treatment efficacy. Likewise, trials conducted after bariatric surgery primarily address altered nutrient absorption and cannot be directly extrapolated to uncomplicated postmenopausal osteoporosis.

Current evidence supports a biological rationale for prebiotic modulation of mineral metabolism, but it does not yet establish a consistent clinical benefit for osteoporosis prevention or treatment. Future trials should evaluate well-defined prebiotic substrates in osteopenic and osteoporotic populations using sufficiently long follow-up periods and clinically relevant skeletal outcomes.

## 7. Probiotics and Synbiotics in the Gut–Bone Axis: Strain-Specific Evidence

### 7.1. Current Clinical Evidence

Probiotics have gained important attention for their ability to modulate microbiota and support bone health. SCFAs are produced by microbial fermentation. In this way, they regulate the immune balance and inhibit osteoclast activity [41]. Some clinical studies have reported favorable changes in bone turnover markers or attenuation of BMD loss with specific probiotic formulations, but these effects are not consistent across strains or populations [18,19].

Products named synbiotics (prebiotics and probiotics) offer synergistic benefits by both introducing beneficial strains and providing substrates for their growth [87]. Compared with the intake of probiotics alone, studies in animal models indicate that synbiotic interventions improve bone microarchitecture and reduce inflammation [88].

Probiotics may influence systemic endocrine pathways. For example, microbial modulation of insulin-like growth factor 1 (IGF-1) may contribute to enhance osteoblast activity and bone formation [41]. A randomized clinical trial by Nilsson et al. (2018) showed that *Lactobacillus reuteri* supplementation reduced bone loss in older women with low bone mineral density [18].

### 7.2. Strain-Specific Clinical Evidence

An important limitation of the current literature is the discussion of probiotics as a homogeneous therapeutic category. Their skeletal effects are likely to be strain specific. For example, *Lactobacillus reuteri* ATCC PTA 6475 has shown beneficial effects on bone loss in older women with low bone mineral density in a randomized clinical trial [18]. By contrast, evidence for other probiotic strains remains largely preclinical and mechanistic, and should not be extrapolated across species or formulations [41]. Future studies should report strain identity precisely, distinguish humans from preclinical evidence, and avoid overgeneralization across probiotic interventions.

Because reported effects vary according to strain, population, endpoint, and follow-up duration, the available evidence is better interpreted through representative quantitative estimates (Table 3).

### 7.3. Recent Randomized Clinical Trials

Recent randomized clinical trials have provided a more detailed picture of the strain- and population-specific effects of probiotic and synbiotic interventions on bone outcomes.

Głogowska-Szeląg et al. evaluated a combination of *Lacticaseibacillus paracasei* LPC100 and *Lactiplantibacillus plantarum* LP140 in postmenopausal women over 12 months. Lumbar spine T-score declined significantly in the placebo group, from 0.06 ± 1.04 to −0.28 ± 1.12 (*p* = 0.041), whereas the decline in the probiotic group, from 0.19 ± 0.99 to −0.08 ± 1.05, was not statistically significant. However, the between-group difference did not reach statistical significance (*p* = 0.089). These findings suggest a possible preservation effect but do not demonstrate an increase in bone mineral density [89].

A randomized controlled study published in 2024 also examined daily *Lactobacillus acidophilus* supplementation in postmenopausal women and assessed calcium status, bone metabolism biomarkers, and bone mineral density. This study provides additional evidence that probiotic effects on skeletal outcomes should be interpreted at the level of the specific strain, intervention duration, and outcome assessed [90].

A 12-month randomized, double-blind, placebo-controlled trial evaluated the synbiotic medical food SBD111 in 286 early postmenopausal women. SBD111 did not significantly reduce lumbar spine bone loss in the overall cohort. Exploratory and prespecified subgroup analyses suggested reduced bone loss at selected skeletal sites among women with osteopenia or higher BMI. These findings illustrate that microbiome-targeted interventions may produce population-specific effects that are not evident in an unselected study population [91].

An important limitation of the current literature is the frequent discussion of probiotics as a homogeneous therapeutic category, although their skeletal effects are likely to be strain specific.

For example, *Lactobacillus reuteri* ATCC PTA 6475 has shown beneficial effects on bone loss in older women with low bone mineral density in a randomized clinical trial [18]. By contrast, evidence for other probiotic strains remains largely preclinical and mechanistic, and should not be extrapolated across species or formulations [41]. Future studies should report strain identity precisely, distinguish humans from preclinical evidence, and avoid overgeneralization across probiotic interventions.

The apparently conflicting findings across randomized trials should be interpreted in the context of substantial biological and methodological heterogeneity. The interventions differed in strain identity and formulation, treatment duration, baseline skeletal status, menopausal stage, and the skeletal outcome measured. Nilsson et al. evaluated *Lactobacillus reuteri* ATCC PTA 6475 in older women with low BMD and assessed tibial volumetric BMD over 12 months [18], whereas Resciniti et al. evaluated a different multi-strain formulation in early postmenopausal women and reported a greater decline in distal tibial volumetric BMD in the probiotic group [69]. Other trials assessed lumbar spine T-scores, bone turnover markers, or composite synbiotic interventions rather than the same skeletal endpoint [19,89,90,91]. Differences in calcium and vitamin D intake, concomitant osteoporosis therapy, baseline microbiome characteristics, adherence, and product viability or formulation may also contribute to variation in treatment response, but these factors were not consistently characterized across studies.

These data indicate that “probiotics” are too biologically heterogeneous to be considered a single therapeutic category without accounting for strain- and formulation-level effects. Meta-analytic estimates that pool different probiotic strains, formulations, populations, and skeletal endpoints should therefore be interpreted cautiously.

The clinical meaning of each endpoint also depends on the population studied. Changes in calcium absorption, CTX, P1NP, or other short-term biomarkers should be interpreted as intermediate outcomes, whereas sustained site-specific changes in BMD provide more clinically relevant structural information. Fragility fracture reduction remains the most clinically meaningful endpoint; however, fracture outcomes have not been adequately evaluated in the available probiotic and synbiotic trials.

It also remains uncertain whether the reported skeletal effects are mediated by measurable remodeling of the gut microbiota or by strain-specific biological activities that may occur independently of major changes in overall microbiome composition.

### 7.4. Safety and Clinical Limitations

Probiotics are generally regarded as safe in the general population. Their use in frail or immunocompromised patients warrants greater caution [92]. This issue is particularly relevant in osteoporosis, which is common in older adults and often coexists with frailty or complex medical comorbidity [93]. In such settings, rare but reported adverse events associated with live biotherapeutic products include bacteremia and other opportunistic infections, particularly in patients with severe underlying disease or impaired host defenses [94].

Fungemia has also been reported in association with probiotic use, particularly with Saccharomyces boulardii in hospitalized or otherwise vulnerable patients [95]. Microbiota-targeted interventions should be individualized and risk-stratified, and not generalized as routine adjunctive therapy in vulnerable patients [92].

Not all clinical studies of microbiota-targeted interventions have shown consistent skeletal benefits [96]. Some randomized trials have reported favorable changes in bone turnover markers, particularly resorption-related markers, without providing equally robust evidence for structural bone outcomes [19]. Other interventional studies have shown neutral findings in the overall study population, despite possible signals of benefit in selected subgroups [91]. Taken together, current human evidence remains heterogeneous, suggesting that treatment response may depend on strain selection, formulation, duration, host characteristics, baseline skeletal status, and the endpoint assessed [96].

## 8. Postbiotics in the Gut–Bone Axis: Definition, Mechanisms, and Emerging Evidence

Postbiotics are a distinct category of microbiome-targeted interventions. Their interpretation in the gut–bone field requires caution. The term is sometimes used broadly for inactivated microbial preparations, microbial structural components, isolated metabolites, or engineered delivery systems, although these categories are not equivalent. This distinction is relevant in osteoporosis research, where many mechanistic studies investigate microbial-derived molecules. For this reason, the evidence should be interpreted according to the exact nature of the intervention.

### 8.1. Terminological Rigor: What Qualifies as a Postbiotic

According to the consensus definition proposed by the International Scientific Association for Probiotics and Prebiotics (ISAPP), a postbiotic is a preparation of inanimate microorganisms and/or their components that confers a health benefit on the host [97].

The definition requires that inactivated microbial cells or their components are present in the final preparation [98].

Purified microbial metabolites administered in isolation do not meet the ISAPP definition of a postbiotic [99]. Isolated short-chain fatty acids, including butyrate, as well as purified bacteriocins or other microbial metabolites administered without accompanying inanimate microbial biomass, should be described according to their specific chemical or functional identity [98].

This distinction is important for the gut–bone literature because some studies described as investigating “postbiotic” effects actually evaluate purified microbial metabolites or isolated bacterial products [99].

Terminological precision is also relevant when evaluating engineered delivery systems. Platforms developed to deliver microbial-derived metabolites may be described as “engineered postbiotics” by individual authors, although such terminology does not indicate compliance with the ISAPP definition [98]. In this review, consensus-defined postbiotics are distinguished from isolated microbial components, purified metabolites, and engineered metabolite-delivery systems.

### 8.2. Mechanistic Rationale for Postbiotics in Bone Metabolism

Microbial structural components can influence bone remodeling through pattern-recognition receptors expressed by immune and skeletal cell lineages. Although isolated bacterial components do not themselves meet the ISAPP definition of a postbiotic, they provide important mechanistic evidence for how components retained within inanimate microbial preparations may affect osteoimmune signaling.

Peptidoglycan-derived signals provide further evidence that microbial structural effects on bone may be receptor-specific and bidirectional. Muramyl dipeptide (MDP), a ligand for NOD2 found in most bacteria, promotes bone formation through Runx2 and β-catenin signaling [100]. By contrast, TriDAP, a NOD1 ligand associated with Gram-negative bacterial peptidoglycan, decreases osteoblast differentiation and Runx2 expression while increasing osteoclast numbers and bone resorption in vivo [100]. This NOD1/NOD2 divergence demonstrates that bacterial structural motifs cannot be considered uniformly osteoprotective.

Sialylated TLR2 has been identified as a receptor for Siglec15 during RANKL-induced osteoclast fusion, providing another mechanism through which TLR2-related signaling can contribute to osteoclast formation [101].

These findings indicate that the skeletal response to microbial structural components depends on the molecular motif involved, the receptor engaged, and the cellular and also the inflammatory context. This distinction is important when interpreting postbiotic strategies for osteoporosis, because the biological activity of an inanimate microbial preparation cannot be inferred simply from its bacterial origin.

### 8.3. Preclinical and Translational Evidence

Recent translational work has extended microbiome-derived therapeutic strategies toward engineered bacterial extracellular vesicles (BEVs). BEVs are nanoscale membrane-bound structures capable of transporting microbial proteins, nucleic acids, and other bioactive molecules and can be modified to improve targeting of specific tissues [102].

Liu et al. developed bioengineered BEVs derived from *Escherichia coli* Nissle 1917 displaying both BMP-2 and CXCR4. The resulting BEVs showed bone-targeting capacity in vivo, promoted osteogenic differentiation of bone marrow mesenchymal stem cells, and significantly prevented bone loss in ovariectomized mice [103]. These findings provide direct preclinical evidence that bacterial vesicle-based platforms can combine microbiome-derived biological signals with targeted skeletal delivery.

Another study, using engineered Akkermansia muciniphila-derived extracellular vesicles has further supported this concept. Bone-targeted vesicles enriched in miR-21-5p accumulated in skeletal tissue, promoted osteogenic differentiation, inhibited osteoclastogenesis, and reduced bone loss in ovariectomized mice through PI3K-AKT signaling [104]. Engineered BEVs may represent a distinct translational extension of microbiome-based therapy for osteoporosis, although they should not be considered equivalent to conventional ISAPP-defined postbiotic preparations.

### 8.4. Current Gaps and Research Priorities

Several gaps currently limit the clinical translation of postbiotic strategies in osteoporosis. Terminology remains inconsistent, and purified microbial metabolites or engineered delivery systems are sometimes described as postbiotics despite not fulfilling the ISAPP consensus definition [97,98]. Most bone-specific evidence remains preclinical, with human studies of well-characterized postbiotic preparations and clinically meaningful skeletal endpoints still scarce. Receptor-specific findings, including the divergent effects of NOD1- and NOD2-mediated signaling, indicate that microbial structural components should not be treated as a homogeneous therapeutic category [100]. Standardization is a major methodological challenge. Current postbiotic preparations differ in microbial composition, inactivation methods, retained cellular structures, and potency, and no single gold-standard quantification method analogous to CFU-based probiotic dosing has been established [99].

Future studies should report the progenitor microorganism, inactivation procedure, retained microbial structures, quantitative characterization, dose metric, and skeletal endpoint in sufficient detail to allow reproducibility and comparison across studies [98].

Engineered bacterial extracellular vesicles and related delivery platforms may broaden the therapeutic potential of microbiome-derived interventions, but they should be evaluated separately from conventional ISAPP-defined postbiotics [103,104].

## 9. Semi-Quantitative Synthesis: Direction and Magnitude of Effects Across Intervention Types

Narrative synthesis alone cannot capture whether the direction of effect is consistent across the accumulating body of RCTs and meta-analyses on microbiome-targeted interventions in osteoporosis, nor whether that consistency varies by intervention type or population. To address this, we constructed a semi-quantitative harvest summary of the most recent meta-analyses and RCTs (2024–2026) reporting bone mineral density (BMD) and bone turnover marker (BTM) outcomes. These findings are summarized in Table 4.

Lumbar spine BMD shows the most recurrent favorable signal across recent meta-analyses. Wang et al. (2024) reported a significant increase in lumbar spine BMD, with an SMD of 0.60 [105]. Wang et al. (2026) also reported a favorable effect at the lumbar spine, with an SMD of 0.85 [107]. Substantial heterogeneity exists, indicating that the magnitude of this effect remains uncertain [107].

Findings for hip BMD and bone turnover markers are less consistent. Wang et al. (2024) reported a significant increase in hip BMD together with reductions in CTX and BALP [105]. In contrast, Wang et al. (2026) found no significant effect on total hip BMD, CTX, osteocalcin, alkaline phosphatase, or osteoprotegerin [107]. This variability likely reflects differences in study populations, probiotic strains, intervention duration, baseline skeletal status, and outcome selection.

A clinically relevant finding was the greater BMD response observed in women with osteopenia compared with those with established osteoporosis [105]. This suggests that probiotic interventions may be more effective in attenuating early bone loss than in reversing established skeletal deficits. The strain-defined randomized trial by Głogowska-Szeląg et al. also showed a pattern more consistent with preservation of lumbar spine bone status than with a clear increase in BMD [89]. These findings support a potential preventive or adjunctive role for microbiome-targeted interventions, not a replacement for established osteoporosis therapy.

Accordingly, the summary was intended to illustrate the direction and approximate magnitude of recently reported effects rather than to rank interventions or establish comparative efficacy across studies. Differences in skeletal site, endpoint definition, effect metric, population, and intervention formulation precluded direct quantitative comparison. No additional statistical pooling, heterogeneity analysis, publication-bias assessment, or independent risk-of-bias evaluation were performed.

The included meta-analyses contain partially overlapping primary trials and should not be interpreted as statistically independent sources of evidence. Differences in probiotic strains, synbiotic formulations, study populations, treatment duration, baseline bone status, and outcome definitions further limit direct comparison across studies.

Apparent agreement in the direction of effect should be interpreted cautiously, particularly when substantial heterogeneity or low certainty of evidence is reported [107].

## 10. Fecal Microbiota Transplantation

Fecal microbiota transplantation (FMT) has been recognized as a radical but increasingly explored approach for correcting gut dysbiosis. When there is a transfer of gut microbiota from a healthy donor into a recipient, FMT can restore the diversity of microbial communities and metabolic function [108]. Preclinical studies indicate that using fecal microbiota transplantation from high bone mass donors have shown results in bone strength and increased SCFA production in recipient mice [109].

Although FMT remains experimental in the context of osteoporosis, it has demonstrated clinical efficacy in recurrent Clostridioides difficile infection [110]. Early data suggest that FMT could be a future therapeutic strategy for osteoporosis, particularly in cases of severe dysbiosis unresponsive to conventional strategies [42].

Despite its mechanistic appeal, FMT should be interpreted with caution in osteoporosis, where the therapeutic risk–benefit threshold differs from that in recurrent Clostridioides difficile infection, the setting in which FMT currently has the strongest clinical support [110].

Osteoporosis is a chronic skeletal disorder for which established antiresorptive and osteoanabolic therapies are already available [111]. In this context, the potential risks of FMT, including pathogen transmission, incomplete long-term safety data, and the complexity of donor screening, may limit its acceptability outside carefully selected research settings [78]. Additional concerns include donor-dependent variability and the lack of standardized approaches for donor selection and product preparation, which may affect both efficacy and reproducibility [108,112]. At present, FMT should be regarded as an experimental strategy in osteoporosis, and not a routine clinical intervention [78]. An additional conceptual limitation in FMT research is the assumption that a universally ‘healthy’ donor microbiota exists [113].

In reality, donor microbial composition varies substantially, and a donor considered healthy by general clinical criteria may not provide a microbial profile optimal for bone-related outcomes [114]. From a gut–bone axis perspective, donor selection may need to consider functionally relevant traits, such as SCFA-producing capacity or enrichment in taxa associated with anti-inflammatory and osteoprotective signaling [112]. Donor-selection strategies remain hypothetical and require validation before clinical implementation [108].

**Figure 3:**
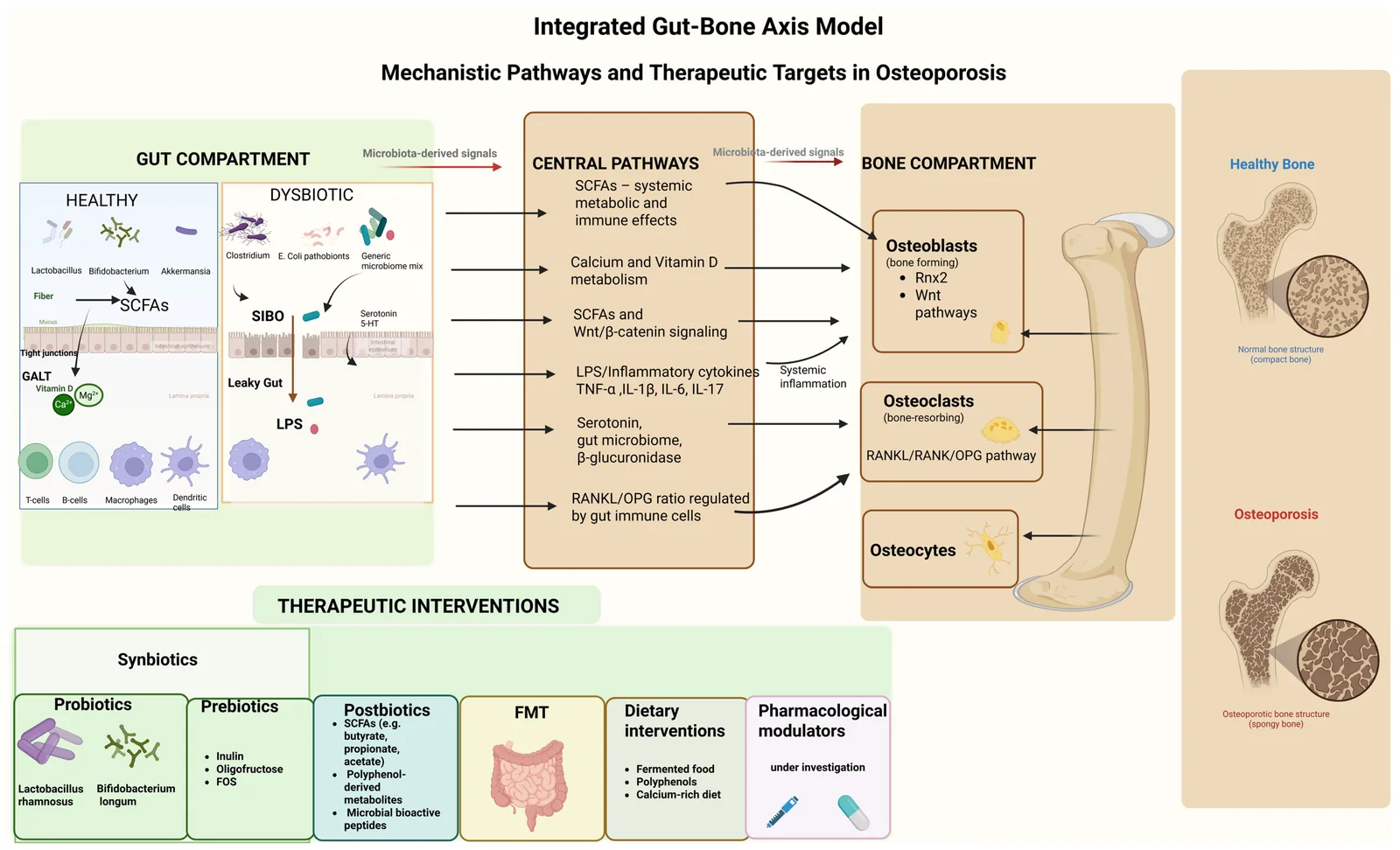
Integrated model of the gut–bone axis showing possible mechanistic pathways and therapeutic targets in osteoporosis. Arrows indicate the proposed direction of interactions among gut microbiota-related mechanisms, immune, metabolic and endocrine pathways, bone remodeling processes, and potential therapeutic interventions; they represent conceptual relationships and do not imply established causality. Created in BioRender. Ispas, S. (2026) https://BioRender.com/9en67hv; accessed on 13 August 2026. **Abbreviations:** FMT, fecal microbiota transplantation; FOS, fructooligosaccharides; GALT, gut-associated lymphoid tissue; IL, interleukin; LPS, lipopolysaccharide; OPG, osteoprotegerin; RANK, receptor activator of nuclear factor kappa-B; RANKL, receptor activator of nuclear factor kappa-B ligand; Runx2, runt-related transcription factor 2; SCFAs, short-chain fatty acids; SIBO, small intestinal bacterial overgrowth; TNF-α, tumor necrosis factor alpha; Wnt, wingless/integrated signaling pathway.

An integrated overview of the gut–bone axis, including gut-related mechanisms, bone remodeling pathways, and potential microbiome-targeted strategies, is shown in Figure 3.

These considerations highlight the importance of diagnosing gut dysbiosis for future clinical decision-making, including assessing osteoporosis risk in patients with dysbiosis or SIBO.

## 11. The Bidirectional Interaction Between Gut Microbiota and Osteoporosis Medications

### 11.1. Why This Axis Matters

An additional clinically relevant but still underexplored aspect of the gut–bone axis is the bidirectional interaction between gut microbiota and conventional osteoporosis medications [115].

Available reviews suggest that gut microbiota may influence drug metabolism, bioavailability, efficacy, and adverse-effect profiles, while pharmacological therapies may in turn modify microbial composition and metabolic output [20]. This perspective is relevant not only to incretin-based approaches, but also to antiresorptive and anabolic therapies used in osteoporosis management [115]. Direct evidence for clinically meaningful microbiota-mediated effects of bisphosphonates, denosumab, or teriparatide remains limited, and the translational relevance of these interactions has not yet been clearly defined [116].

### 11.2. Drug-Induced Osteoporosis and Microbiota Alterations

Drug-induced osteoporosis provides an important context in which medication exposure, skeletal deterioration, and gut microbiota alterations may occur simultaneously. Martiniakova et al. reviewed the available evidence across several drug classes associated with secondary osteoporosis, including glucocorticoids, aromatase inhibitors, proton pump inhibitors, antiretroviral agents, antiepileptic drugs, antipsychotics, and antidepressants [21]. For most of these drug classes, direct evidence demonstrating that drug-induced changes in the gut microbiota mediate alterations in bone turnover, BMD, or fracture risk remains limited or absent [21]. In many studies, microbial and skeletal changes have been documented in parallel. This distinction is important because an association between a medication, microbiota composition, and bone loss does not establish microbiota-mediated skeletal toxicity.

### 11.3. Osteoporosis Treatments as Modifiers of the Gut Microbiota

Evidence for the reverse direction of the relationship is considerably more limited. Deng et al. compared teriparatide and denosumab in patients with osteoporotic fractures and assessed BMD, bone turnover markers, and fecal microbiota before and after six months of treatment [117]. Both therapies increased lumbar spine and hip BMD but produced different patterns of bone turnover and distinct alterations in gut microbial composition [117]. Changes in microbial profiles were also associated with bone-related indicators, including pathways related to short-chain fatty acid metabolism [117]. These findings provide clinical evidence that osteoporosis pharmacotherapy itself may modify the gut microbiota, although the study design does not establish that these microbial changes mediate the skeletal response to treatment.

### 11.4. Can Microbiota Modify Treatment Response?

A less explored question is whether baseline gut microbiota composition or treatment-induced microbial changes influence the magnitude of response to antiresorptive or anabolic osteoporosis therapy. Current human evidence is insufficient to establish the gut microbiota as a validated predictor of BMD response, fracture reduction, or treatment tolerability. Available studies of treatment response have primarily evaluated conventional clinical predictors, such as baseline BMD, age, bone turnover markers, or previous osteoporosis therapy, and not microbiota-derived biomarkers.

This represents a major unresolved component of the bidirectional microbiota–drug–bone relationship. Prospective studies should evaluate whether baseline microbial signatures, microbial metabolites, or treatment-induced microbiota changes independently predict skeletal response to osteoporosis pharmacotherapy.

At present, microbiome-targeted interventions should be considered investigational adjunctive or preventive strategies rather than alternatives to established antiresorptive or osteoanabolic therapies. Evidence is currently insufficient to support replacement of standard osteoporosis treatment with probiotics, prebiotics, synbiotics, or postbiotics. Their potential use alongside conventional therapy requires further validation regarding efficacy, safety, and treatment interactions.

## 12. Microbiota-Related Biomarkers in Osteoporosis: Current Validation Status

### 12.1. Microbial Taxa as Candidate Biomarkers

Selected SCFA-producing taxa have been proposed as candidate microbiota-related biomarkers of skeletal health. *Faecalibacterium prausnitzii* is a potential candidate because of its anti-inflammatory properties and association with butyrate production [67]. Its diagnostic or prognostic value in osteoporosis has not been prospectively validated.

### 12.2. Microbial Metabolites as Candidate Biomarkers

Microbial metabolites may provide a more functional assessment of the gut–bone axis than taxonomic profiling alone. Short-chain fatty acids, particularly butyrate, have been associated with osteoprotective mechanisms and may represent candidate translational biomarkers [31]. Clinically meaningful thresholds and standardized analytical approaches have not been established.

### 12.3. Intestinal Barrier and Inflammatory Biomarkers

Circulating markers of intestinal barrier dysfunction and endotoxemia-related inflammation may complement microbial and metabolite profiling. Impaired intestinal barrier integrity and chronic low-grade inflammation are plausible mediators linking dysbiosis to bone loss, but the diagnostic performance and clinical thresholds of these markers in osteoporosis remain undefined [118].

### 12.4. Current Clinical Readiness

At present, no microbiota-related biomarker panel has been standardized or prospectively validated for osteoporosis [67]. Available microbial taxa, metabolites, and host-response markers should be considered hypothesis-generating research tools. Future studies should determine whether such biomarkers improve risk stratification or prediction of treatment response beyond established clinical and densitometric parameters.

## 13. Limitations

Several challenges limit the clinical uptake of microbiome research, despite promising results.

First, generalizing findings is difficult, given the interindividual variability in microbiota composition. Microbial communities are shaped by factors such as age, sex, diet, geography, and comorbidities, which complicates the design of universal interventions [9].

Second, human studies often rely on observational data that establish associations, but not causality. Moreover, even though animal models do provide mechanistic insights, they cannot fully capture the complexities of bone physiology or the diversity of human microbiota [26].

In addition, germ-free models, although mechanistically informative, do not fully recapitulate the partial dysbiosis and environmental complexity typical of human disease [119]. Longitudinal, randomized controlled trials (RCTs) are still limited, given that the existing ones are generally limited by small sample sizes and short follow-up periods.

Third, an obstacle continues to be the standardization of interventions. Not all probiotic strains provide bone benefits, since probiotic effects are strain specific. Moreover, FMT protocols raise safety and efficacy concerns, as they lack uniformity in terms of donor selection, preparation, and delivery. This is particularly relevant in osteoporosis, where FMT remains investigational [78]. The regulatory frameworks for microbiome-based therapeutics are still evolving, which creates additional barriers to clinical application [41].

Finally, clarification is needed regarding the interaction with existing therapies. Many patients with osteoporosis already take antiresorptive or anabolic drugs, and the synergistic/antagonistic effects of microbiome-based therapies with these medications require additional investigation.

## 14. Discussion

This narrative review integrates data from the literature supporting the existence of the gut–bone axis [3]. But how does this relate to clinical outcomes? Current data demonstrate the complexity of biological mechanisms involved and the interaction between microbiota, immune responses, and endocrine pathways [54]. This review describes the ways in which gut dysbiosis influences bone metabolism [67]. Alterations in the microbiota are associated with impaired mineral absorption [32], chronic low-grade inflammation [8], and dysregulation of osteoimmune signaling [35]. Thus, all these changes may lead to an imbalance between bone resorption and formation, favoring osteolysis.

From an osteoimmunological perspective, gut dysbiosis may promote systemic immune activation. Increased levels of proinflammatory cytokines have been implicated in promoting osteoclastogenesis [43]. The imbalance between Th17 cells and regulatory T cells appears to be a common mechanism of osteoporosis and senescence, and gut inflammation may play an important role in bone loss [44]. These observations are consistent with existing literature. All these findings suggest that the microbiota acts as a modulator of the immune-bone axis.

The integration of endocrine signaling within the gut–bone axis represents another aspect that we have analyzed. A recent review has further expanded this framework toward a gut–brain–bone axis, emphasizing the potential contribution of neuroendocrine signaling to microbiota–bone interactions. Shi et al. described a regulatory network linking gut dysbiosis, microbial metabolites, central nervous system signaling, the hypothalamic–pituitary–adrenal axis, gut hormones, and skeletal remodeling. This broader model suggests that stress-related and neuroendocrine pathways may interact with immune and metabolic mechanisms already implicated in the gut–bone axis. However, much of this framework remains mechanistic, and its direct clinical relevance to osteoporosis requires further validation in prospective human studies [120]. Bile acid metabolism has been associated with bone mineral density in postmenopausal women [121]. Microbiota-related signaling may also interact with the secretion of gut hormones, such as GIP, GLP-1, and GLP-2. These gut hormones can exert direct and indirect effects on bone remodeling [56]. A detailed study of these mechanisms will expand the classical perspective on osteoporosis. This suggests that bones are sensitive to endocrine signals influenced by the microbiota.

We analyzed the data from the literature and found that SIBO may be associated with nutritional and body-composition abnormalities relevant to skeletal health. Clinical evidence linking SIBO to reduced bone mineral density remains limited [73]. Nutrient malabsorption, systemic inflammation, and enteroendocrine dysfunction are mechanisms by which SIBO may contribute to skeletal fragility. In our opinion, more data is needed, and we believe that an evaluation of the digestive tract should be considered in patients with unexplained osteoporosis.

A clinically feasible microbiota-related biomarker panel for osteoporosis has not yet been standardized or prospectively validated. Current open-access reviews consistently indicate that microbiota-associated skeletal biomarkers remain investigational [67].

Recurrent signals in the literature suggest that a pragmatic candidate panel could include fecal short-chain fatty acid profiling, particularly butyrate-related signatures. SCFAs, especially butyrate, are described as central mediators of gut–bone signaling and are linked to osteoprotective mechanisms across current reviews [31].

Selected SCFA-producing taxa may also be considered candidate components of such a panel. In particular, *Faecalibacterium prausnitzii* is discussed in recent open-access osteoporosis reviews as a potentially protective taxon because of its anti-inflammatory and butyrate-associated profile, although its value as a clinical biomarker remains unvalidated [67].

A translational panel could also incorporate circulating markers related to intestinal barrier dysfunction or endotoxemia-associated inflammation, because current gut–bone reviews consistently describe impaired barrier integrity and low-grade inflammation as plausible mediators linking dysbiosis to bone loss. The diagnostic performance and clinical thresholds of such markers in osteoporosis remain undefined [118].

Any proposed microbiota-related biomarker panel should currently be regarded as hypothesis-generating and research-oriented because they are not validated tools for routine osteoporosis workup [67].

From a translational perspective, dietary fiber modifications [122], prebiotics, probiotics [87], and fecal microbiota transplantation [66] aim to target new biological mechanisms of osteoporosis. Although early findings are promising, large-scale randomized clinical trials are needed to define the efficacy and personalization of these strategies. Despite compelling mechanistic evidence, causality in humans remains difficult to establish. A more rigorous causal framework will require converging evidence from complementary human study designs [119]. Prospective longitudinal cohorts with repeated microbiome, metabolite, and skeletal assessments are needed to determine whether microbial alterations precede bone loss [119]. Genetically informed approaches, including Mendelian randomization, may help clarify potentially causal links between microbial traits, host metabolic mediators, and bone-related outcomes [53]. Twin designs may reduce confounding related to host genetics and shared early-life exposures [123]. Adequately powered interventional trials with validated skeletal endpoints and mechanistic biomarkers are required to test whether targeted microbiota modulation can reproducibly influence bone turnover, mineral metabolism, and bone mineral density [119]. Interindividual variability in microbiota composition limits the generalizability of current findings.

In conclusion, the gut–bone axis represents a current conceptual framework debated in the literature. In our opinion, it could provide an integrative perspective on the pathogenesis of osteoporosis. Understanding the interactions between the microbiota, the immune system, and endocrine signaling could open new directions for the prevention and personalized management of bone diseases.

## 15. Future Directions

After analysing the literature, we observed rapid scientific progress in gut–bone axis research. While recent research is promising, key questions remain unanswered. For example, how does modulating the microbiome influence bone outcomes?

We identified that few studies exist analyzing how the microbiome influences bone density according to gender and different age groups. Future reviews should also cover the gaps in the existing database and focus on:

1. standardization of microbiome assessment methods and interpretation of results;
2. large longitudinal cohort studies evaluating microbiome–bone interactions;
3. randomized controlled trials assessing specific prebiotic, probiotic, synbiotic, and postbiotic interventions in bone diseases;
4. development of standardized SIBO diagnostic protocols and interventional studies evaluating bone outcomes;
5. evaluation of whether strain-specific probiotic supplementation can reduce bone loss in well-defined osteopenic or osteoporotic populations using validated skeletal endpoints and sufficiently long follow-up;
6. assessment of whether baseline microbiota composition or SCFA-related signatures predict differential response to probiotic, synbiotic, prebiotic, or dietary interventions;
7. identification of whether patients with unexplained osteoporosis and gastrointestinal symptoms represent a subgroup more likely to benefit from targeted evaluation and treatment of SIBO or other forms of dysbiosis;
8. validation of microbiota-related biomarkers for improving risk stratification or treatment selection beyond conventional osteoporosis assessment tools;
9. investigation of whether gut microbiota influences the efficacy or tolerability of standard osteoporosis medications, including antiresorptive and anabolic therapies.

Further studies are needed to investigate in greater depth the role of the microbiome as a biomarker in different pathologies. A deeper understanding of these mechanisms may contribute to the development of personalized, patient-centred medicine.

## 16. Conclusions

Current evidence suggests that gut microbiota may contribute to skeletal health through effects on nutrient absorption, osteoimmune regulation, microbial metabolites, and endocrine signaling. In this way, metabolic-immune-endocrine pathways contribute to bone remodeling and skeletal integrity.

From a clinical perspective, alterations in gut microbiota composition may be associated with reduced bone mineral density and increased osteoporosis risk. These findings support further investigation of microbiome-targeted strategies, including dietary interventions, prebiotics, probiotics, synbiotics, postbiotics, and fecal microbiota transplantation, as potential adjunctive approaches to conventional osteoporosis therapies. These approaches should not currently be considered substitutes for established osteoporosis pharmacotherapy.

Future research should focus on developing biomarkers and interventions targeting the gut–bone axis, as well as on expanding randomized controlled clinical trials and application of precision medicine approaches. Intervention adaptation should be based on the microbial and host profile. The gut–bone axis should currently be viewed as an emerging translational framework.

## Figures and Tables

**Table 1:** Evidence categories used to interpret studies examining the gut–bone axis in osteoporosis.

Evidence Category	Examples in Gut–Bone Research	Main Outcomes Assessed	What the Evidence Can Support	Main Limitations	Representative Evidence
Experimental evidence	Germ-free animals, ovariectomized models, antibiotic-induced dysbiosis, metabolite supplementation	Bone microarchitecture, osteoclast activity, cytokines, mineral absorption	Mechanistic plausibility and causal effects under controlled conditions	Limited generalizability to humans	[12,13,14]
Cross-sectional and case–control human studies	Comparisons among normal BMD, osteopenia, and osteoporosis	Microbial diversity, taxa abundance, BMD, bone turnover markers	Associations and hypothesis generation	Reverse causality, residual confounding, inconsistent microbial signatures	[15,16]
Longitudinal human studies	Cohorts relating microbiome profiles to subsequent bone loss or fracture	Change in BMD, incident osteoporosis, fragility fractures	Temporality and possible prognostic value	Few studies, heterogeneous methods, limited fracture events	[17]
Human intervention studies	Trials of probiotics, prebiotics, synbiotics, diet, or microbial metabolites	Bone turnover markers, calcium absorption, BMD, safety	Preliminary evidence of intervention effects	Small samples, short duration, strain-specific effects, limited replication	[18,19]

Note: These categories are used to support interpretation of the literature and do not represent a formal evidence-grading system. Clinical relevance depends on both study design and the outcome assessed; incident fractures and sustained changes in bone mineral density are more clinically meaningful than changes in microbial composition or bone turnover markers alone.

**Table 2:** Gut-derived hormones and microbiota-related signals involved in bone remodeling.

Hormone	Receptors/Cellular Targets	Main Skeletal Effects	Key Signaling Pathways	Supporting Evidence
GIP	GIPR are expressed on osteoblasts, osteocytes, and osteoclasts	Enhances osteoblast survival and activity. Suppresses osteoclastogenesis and induces osteoclast apoptosis. Reduces bone resorption.	NF-κB, Src, Akt, and p38 MAPK. These pathways lead to impaired nuclear translocation of nuclear factor of activated T cells-1 (NFATc1) and nuclear factor-κB (NF-κB).	[57,59,60,62]
GLP-1	GLP-1R is expressed on osteoblasts and stromal cells	Promotes osteoblast proliferation/differentiation. Decreases osteoclast activity.	PKA, MAPK, PI3K	[56,60]
GLP-2	GLP-2R not clearly demonstrated in mature human bone cells Intestinal-PTH axis-mediated effects	Reduces bone resorption. May increase BMD.	Indirect, intestine-mediated; possible intestine-PTH axis-mediated effects	[60]
PYY	Y1R are located on osteoblasts, mainly in murine models, with limited evidence in human bone cells	Secondary bone effects; Indirect skeletal targets	Y-receptors-mediated pathways	[60]
Microbiota-derived SCFAs	Enteroendocrine cells L and K cells	Stimulate secretion of incretins (GIP/GLP-1/GLP-2); modulate remodeling indirectly	GPCR-mediated enteroendocrine signaling	[56,63]

Abbreviations: GIP, glucose-dependent insulinotropic polypeptide; GLP-1, glucagon-like peptide-1; GLP-2, glucagon-like peptide-2; PYY, peptide YY; GIPR, glucose-dependent insulinotropic polypeptide receptor; GLP-1R, glucagon-like peptide-1 receptor, GLP-2R, glucagon-like peptide-2 receptor; Y1R, neuropeptide Y receptor type 1; SCFAs, short-chain fatty acids; NF-κB, nuclear factor kappa B; NFATc1, nuclear factor of activated T cells 1; MAPK, mitogen-activated protein kinase; PI3K, phosphoinositide 3-kinase; Akt, protein kinase B; PKA, protein kinase A; Src, proto-oncogene tyrosine-protein kinase Src; p38 MAPK, p38 mitogen-activated protein kinase, GPCR, G protein-coupled receptor, BMD, bone mineral density; PTH, parathyroid hormone.

**Table 4:** Semi-quantitative harvest summary of recent randomized trials and meta-analyses evaluating microbiome-targeted interventions and skeletal outcomes.

Study	Design and Population	Intervention	Outcome Type	Main Findings	Interpretation	Reference
Wang et al., 2024	Systematic review and meta-analysis; 12 RCTs; 1183 postmenopausal women	Probiotics	BMD + BTMs	Lumbar spine BMD: SMD 0.60 (95% CI 0.14–1.05); hip BMD: SMD 0.74 (95% CI 0.15–1.33); CTX: SMD −1.51; BALP: SMD −1.80	Favorable effects on lumbar and hip BMD and selected BTMs; greater BMD benefit in women with osteopenia	[105]
Głogowska-Szeląg et al., 2024	Multicenter randomized placebo-controlled trial; 167 postmenopausal women; 12 months	*Lacticaseibacillus paracasei* LPC100 + *Lactiplantibacillus plantarum* LP140	BMD	Lumbar spine T-score remained stable in the probiotic group, while a significant decline occurred in placebo	Suggests BMD preservation, not clear BMD gain	[89]
Yuan et al., 2026	Meta-analysis; 15 RCTs; 1432 middle-aged and older participants with osteoporosis	Probiotics	BTMs	P1NP: MD +8.4 μg/L; CTX-I: SMD −0.35; osteocalcin also increased	Supports favorable effects on selected bone formation and resorption markers	[106]
Wang et al., 2026	Meta-analysis; 10 RCTs; 732 participants with osteoporosis	Probiotics and/or synbiotics	BMD + BTMs	Lumbar spine BMD: SMD 0.85; PTH: SMD −1.21; no significant total hip BMD effect	Favorable lumbar-spine signal, but substantial heterogeneity and low certainty	[107]

## Data Availability

No new data were generated or analyzed in this review. Data sharing is not applicable to this article.

## Supplementary Materials

The following supporting information can be downloaded at: https://www.mdpi.com/article/doi/s1, Table S1. PubMed, Scopus, and Web of Science search strategies used for each thematic section of the review; Table S2. Inclusion and exclusion criteria.

## Abbreviations

The following abbreviations are used in this manuscript:

AhR	aryl hydrocarbon receptor
ALP	alkaline phosphatase
AMPK	AMP-activated protein kinase
Ca^2+^	calcium
BIA	bioelectrical impedance analysis
BMD	bone mineral density
BMMSCs	bone marrow mesenchymal stem cells
BMP2	bone morphogenetic protein 2
cAMP	cyclic adenosine monophosphate
DCs	dendritic cells
E-cadherin	epithelial cadherin
ERK	extracellular signal-regulated kinase
FXR	farnesoid X receptor
FMT	faecal microbiota transplantation
GALT	gut-associated lymphoid tissue
GH	growth hormone
GIP	glucose-dependent insulinotropic polypeptide
GIPR	glucose-dependent insulinotropic polypeptide receptor
GLP-1	glucagon-like peptide-1
GLP-1R	glucagon-like peptide-1 receptor
GLP-2	glucagon-like peptide-2
GLP-2R	glucagon-like peptide-2 receptor
GPCR	G-protein-coupled receptor
HDAC	histone deacetylase
IAA	indole-3-acetic acid
IFN	interferon
IGF-1	insulin-like growth factor-1
IL	interleukin
ILC	innate lymphoid cell
ILC1	innate lymphoid cell type 1
ILC3	innate lymphoid cell type 3
IPA	indole-3-propionic acid
IRF	interferon regulatory factor
LCA	lithocholic acid
LPS	lipopolysaccharide
MAPK	mitogen-activated protein kinase
Mg^2+^	magnesium
MSC	mesenchymal stem cell
MyD88	myeloid differentiation primary response protein 88
NF-κB	nuclear factor kappa B
NFATc1	nuclear factor of activated T cells 1
OPG	osteoprotegerin
PAMPs	pathogen-associated molecular patterns
P1NP	procollagen type 1 *N*-terminal propeptide
PTH	parathyroid hormone
PRISMA	Preferred Reporting Items for Systematic Reviews and Meta-Analyses
PYY	peptide YY
RANKL	receptor activator of nuclear factor κB ligand
RXR	retinoid X receptor
SCFAs	short-chain fatty acids
SIBO	small intestinal bacterial overgrowth
Src	proto-oncogene tyrosine-protein kinase Src
Th	T helper cell
Th1	T helper 1 cell
Th17	T helper 17 cell
TLRs	Toll-like receptors
TGR5	Takeda G-protein-coupled bile acid receptor 1
TNF α	tumor necrosis factor α
Trp	tryptophan
TRIF	TIR-domain-containing adapter-inducing interferon-β
Tregs	regulatory T cells
VDR	vitamin D receptor
Wnt	Wingless-related integration site protein
ZO-1	Zonula occludens-1
