# Supplementary material for: Targeting the Gut–Bone Axis with Prebiotics, Probiotics, Synbiotics, and Postbiotics: From Mechanistic Plausibility to Clinical Bone Outcomes
Source: Nutrients. 2026 Sep 10;18(18):2967. doi: 10.3390/nu18182967 (PMC13610306; doi:10.3390/nu18182967)
Supplement: Supplementary file 1 [file nutrients-18-02967-s001.zip › Supplementary Table S1.pdf]

Supplementary Table S1. PubMed, Scopus, and Web of Science search strategies used for each thematic section of the review

Strategy/Section	Source / tool	Search approach	Search concepts	Filters / notes
Mechanisms & dysbiosis–osteoporosis evidence	PubMed	MeSH<br>title/abstract [tiab]	("Osteoporosis"[MeSH] OR osteoporosis[tiab] OR osteopenia[tiab] OR "bone mineral density"[tiab] OR "bone remodeling"[tiab] OR "bone loss"[tiab]) AND ("gut microbiota"[tiab] OR "gut microbiome"[tiab] OR dysbiosis[tiab] OR "gut-bone axis"[tiab] OR "short-chain fatty acid*"[tiab] OR SCFA*[tiab] OR butyrate[tiab] OR "bile acid*"[tiab] OR FXR[tiab] OR TGR5[tiab] OR tryptophan[tiab] OR "aryl hydrocarbon receptor"[tiab])	English; 2021–2026; articles and reviews. Earlier seminal studies published before 2021 were additionally included when directly relevant to the conceptual, mechanistic, or clinical framework
	Scopus	TITLE-ABS-KEY	((osteoporosis OR osteopenia OR "bone mineral density" OR "bone remodeling" OR "bone loss") AND ("gut microbiota" OR "gut microbiome" OR dysbiosis OR "gut-bone axis" OR "short-chain fatty acid*" OR SCFA* OR butyrate OR "bile acid*" OR FXR OR TGR5 OR tryptophan OR "aryl hydrocarbon receptor"))	English; 2021–2026; articles and reviews. Earlier seminal studies published before 2021 were additionally included when directly relevant to the conceptual, mechanistic, or clinical framework
	Web of Science Collection	TS=	((osteoporosis OR osteopenia OR "bone mineral density" OR "bone remodeling" OR "bone loss") AND ("gut microbiota" OR "gut microbiome" OR dysbiosis OR "gut-bone axis" OR "short-chain fatty acid*" OR SCFA* OR butyrate OR "bile acid*" OR FXR OR TGR5 OR tryptophan OR "aryl hydrocarbon receptor"))	English; 2021–2026; articles and reviews. Earlier seminal studies published before 2021 were additionally included when directly relevant to the conceptual, mechanistic, or

				clinical framework
Microbiome-targeted interventions: prebiotics, probiotics, synbiotics, postbiotics, FMT	PubMed	MeSH; title/abstract [tiab]	("Osteoporosis"[MeSH] OR osteoporosis[tiab] OR osteopenia[tiab] OR "bone mineral density"[tiab] OR "bone turnover"[tiab]) AND (prebiotic*[tiab] OR probiotic*[tiab] OR synbiotic*[tiab] OR postbiotic*[tiab] OR "fecal microbiota transplant*"[tiab] OR "extracellular vesicle*"[tiab])	English; 2021–2026; articles and reviews. Earlier seminal studies published before 2021 were additionally included when directly relevant to the conceptual, mechanistic, or clinical framework
	Scopus	TITLE-ABS-KEY	((osteoporosis OR osteopenia OR "bone mineral density" OR "bone turnover") AND (prebiotic* OR probiotic* OR synbiotic* OR postbiotic* OR "fecal microbiota transplant*" OR "extracellular vesicle*")) English; 2020–2026; articles and reviews.	English; 2021–2026; articles and reviews. Earlier seminal studies published before 2021 were additionally included when directly relevant to the conceptual, mechanistic, or clinical framework
	Web of Science Collection	TS=	((osteoporosis OR osteopenia OR "bone mineral density" OR "bone turnover") AND (prebiotic* OR probiotic* OR synbiotic* OR postbiotic* OR "fecal microbiota transplant*" OR "extracellular vesicle*"))	English; 2021–2026; articles and reviews. Earlier seminal studies published before 2021 were additionally included when directly relevant to the conceptual, mechanistic, or clinical framework
Small intestinal bacterial overgrowth	PubMed	MeSH, title/abstract [tiab]	("small intestinal bacterial overgrowth"[tiab] OR SIBO[tiab]) AND (osteoporosis[tiab] OR "bone"[tiab] OR "breath test"[tiab] OR diagnos*[tiab])	English; 2021–2026; articles and reviews. Earlier seminal studies published before 2021 were additionally included when

				directly relevant to the conceptual, mechanistic, or clinical framework
	Scopus	TITLE-ABS-KEY	(("small intestinal bacterial overgrowth" OR SIBO) AND (osteoporosis OR bone OR "breath test" OR diagnos*))	English; 2021–2026; articles and reviews. Earlier seminal studies published before 2021 were additionally included when directly relevant to the conceptual, mechanistic, or clinical framework
	Web of Science Collection	TS=	(("small intestinal bacterial overgrowth" OR SIBO) AND (osteoporosis OR bone OR "breath test" OR diagnos*))	English; 2021–2026; articles and reviews. Earlier seminal studies published before 2021 were additionally included when directly relevant to the conceptual, mechanistic, or clinical framework
Bidirectional drug–microbiota interaction	PubMed	MeSH, title/abstract [tiab]	("drug-induced osteoporosis"[tiab] OR "secondary osteoporosis"[tiab] OR teriparatide[tiab] OR denosumab[tiab] OR pharmacomicrobiomics[tiab]) AND ("gut microbiota"[tiab] OR "gut microbiome"[tiab] OR dysbiosis[tiab])	English; 2021–2026; articles and reviews. Earlier seminal studies published before 2021 were additionally included when directly relevant to the conceptual, mechanistic, or clinical framework
	Scopus	TITLE-ABS-KEY	(("drug-induced osteoporosis" OR "secondary osteoporosis" OR teriparatide OR denosumab OR pharmacomicrobiomics) AND ("gut microbiota" OR	English; 2021–2026; articles and reviews. Earlier seminal studies published before

			"gut microbiome" OR dysbiosis))	2021 were additionally included when directly relevant to the conceptual, mechanistic, or clinical framework
	Web of Science Collection	TS=	(("drug-induced osteoporosis" OR "secondary osteoporosis" OR teriparatide OR denosumab OR pharmacomicrobiomics) AND ("gut microbiota" OR "gut microbiome" OR dysbiosis))	English; 2021–2026; articles and reviews. Earlier seminal studies published before 2021 were additionally included when directly relevant to the conceptual, mechanistic, or clinical framework
Safety of probiotic/synbiotic interventions	PubMed	MeSH; title/abstract [tiab]	(probiotic*[tiab] OR synbiotic*[tiab] OR "Probiotics"[MeSH]) AND (safety[tiab] OR bacteremia[tiab] OR fungemia[tiab] OR "adverse event*"[tiab] OR immunocompromised[tiab] OR frail*[tiab])	English; 2021–2026; articles and reviews. Earlier seminal studies published before 2021 were additionally included when directly relevant to the conceptual, mechanistic, or clinical framework
	Scopus	TITLE-ABS-KEY	((probiotic* OR synbiotic*) AND (safety OR bacteremia OR fungemia OR "adverse event*" OR immunocompromised OR frail*))	English; 2021–2026; articles and reviews. Earlier seminal studies published before 2021 were additionally included when directly relevant to the conceptual, mechanistic, or clinical framework
	Web of Science Collection	TS=	((probiotic* OR synbiotic*) AND (safety OR bacteremia OR fungemia OR "adverse	English; 2021–2026; articles and reviews.

			event*" OR immunocompromised OR frail*))	Earlier seminal studies published before 2021 were additionally included when directly relevant to the conceptual, mechanistic, or clinical framework

Note: Strategies A1–A5 were developed separately, one per thematic sub-question of the review, because a single combined bone-condition AND microbiota strategy failed to retrieve methodological or safety-related references that do not explicitly mention bone-related terms. Each strategy was validated by searching the first author's surname of a reference already known to belong to that section, alongside the strategy's main terms. Earlier seminal studies published before 2020 were additionally included when directly relevant to the conceptual, mechanistic, or clinical framework of the gut–bone axis. Last search date: 10 August 2026.
