# Supplementary material for: Targeting the Gut–Bone Axis with Prebiotics, Probiotics, Synbiotics, and Postbiotics: From Mechanistic Plausibility to Clinical Bone Outcomes
Source: Nutrients. 2026 Sep 10;18(18):2967. doi: 10.3390/nu18182967 (PMC13610306; doi:10.3390/nu18182967)
Supplement: Supplementary file 1 [file nutrients-18-02967-s001.zip › Supplementary Table S2.pdf]

Supplementary Table S2. Inclusion and exclusion criteria

**Category**	**Inclusion criteria**	**Exclusion criteria**
Topic relevance	Studies addressing the gut microbiota, gastrointestinal microbiome, dysbiosis, small intestinal bacterial overgrowth (SIBO), or the gut-bone axis.	Studies not related to gut microbiota, dysbiosis, SIBO, or the gut–bone axis.
Bone-related outcomes	Studies reporting or discussing bone mineral density, osteoporosis, osteopenia, bone turnover markers, osteoblast/osteoclast activity, bone remodeling, bone loss, bone metabolism, or fracture risk.	Studies without skeletal outcomes or without mechanistic relevance to bone metabolism.
Study type	Human studies, animal studies, experimental studies, translational studies, clinical and observational studies, narrative reviews, systematic reviews, and meta-analyses.	Conference abstracts without full text, letters, comments, editorials without original or mechanistic discussion, comments, and opinion pieces.
Mechanistic relevance	Studies addressing mechanisms relevant to the gut-bone axis, including short-chain fatty acids, bile acids, tryptophan metabolites, intestinal permeability, osteoimmunology, mineral absorption, endocrine signaling, incretin hormones, inflammation, or microbiota-derived metabolites.	Studies mentioning microbiota or osteoporosis only superficially, without mechanistic, translational, or clinical relevance to the gut–bone axis.
Therapeutic/preventive relevance	Studies investigating microbiome-targeted or microbiome-related interventions, including diet, fiber, prebiotics, probiotics, synbiotics, postbiotics, fermented foods, fecal microbiota transplantation, or pharmacological approaches with relevance to the gut–bone axis. Studies addressing drug-induced microbiota alterations, the effects of osteoporosis medications on gut microbiota, or microbiota-related biomarkers were also considered.	Studies focused on interventions unrelated to microbiota, dysbiosis, SIBO, or bone health.
Population/model	Studies involving humans, animal models, in vitro models, or experimental systems relevant to bone metabolism and gut microbiota.	Studies conducted in models or populations with no relevance to skeletal health or gut microbiota-mediated mechanisms.
Language	Articles published in English.	Articles not published in English.
Publication period	Articles published between 2021 and 2026 were prioritized. Earlier seminal studies were included when directly relevant to the conceptual,	Older articles were excluded if they were not foundational, not mechanistically relevant, or duplicated by more recent evidence.

	mechanistic,translational or clinical framework of the gut–bone axis.	
Availability	Full-text articles available for assessment.	Articles for which the full text was not available.
Duplication	The most complete or most relevant version of overlapping evidence was considered.	Duplicate records or overlapping publications without additional relevant information.
